# Editorial: Age differences in trust and fraud

**DOI:** 10.3389/fpsyg.2024.1455499

**Published:** 2024-08-08

**Authors:** Lixia Yang, Xin Zhang, Jing Yu

**Affiliations:** ^1^Department of Psychology, Toronto Metropolitan University, Toronto, ON, Canada; ^2^School of Psychological and Cognitive Science, Peking University, Peking, China; ^3^Department of Psychology, Southwest University, Chongqing, China

**Keywords:** aging, trust, fraud, editorial, social cognition

Fraud can be a serious social problem nowadays (e.g., Ross et al., 2014), with detrimental financial and health consequences that may severely affect older adults (Button et al., 2014; Kircanski et al., 2018). Anecdotally and empirically, older adults are hypothesized to be more vulnerable to fraud victimization than younger adults (Carcach et al., 2001). Although age differences in fraud victimization have been overwhelmingly featured in news and media reports, empirical research did not reach a consensus. On the one hand, some research supports an increased fraud victimization susceptibility in older adults. For example, among community-dwelling older adults, older age is associated with heightened susceptibility to financial victimization (James et al., 2012). Furthermore, older adults' cognitive declines make them more likely to be subject to false memory, distraction and interference, and slower processing (e.g., Jacoby and Rhodes, 2006), which might contribute to their poor financial decision-making (Han et al., 2015; Wilson et al., 2016; Ebner et al., 2020). In support of this, past research did identify cognitive ability as a crucial predictor of fraud victimization among older adults (Judges et al., 2017). Additionally, there are also some personality and social factors for heightened fraud vulnerability in older adults, such as lower honesty and humility, lower conscientiousness, higher social isolation and loneliness, and lower self-control (Gottfredson and Hirschi, 1990; Alves and Wilson, 2008; Judges et al., 2017).

On the other hand, it has also been suggested that aging is associated with some protective factors that shield older adults against scam victimization. For example, older adults' higher level of emotional understanding might help them to correctly identify emotional warning signs and thus reduce their susceptibility to scams (Mueller et al., 2020). Furthermore, older adults are differentially more resistant to persuasion and more sensitive to risks, which might protect them from scam responding (Rolison et al., 2019). However, little is known about the underlying neural and biological mechanisms, as well as the psychological and individual profiles of aging fraud victims.

In this Research Topic, a collection of articles features research findings that fill some of the aforementioned gaps in the literature. Shang et al. reported a systematic review of internet fraud victimization among older adults. Previous studies revealed some psychosocial determinants/characteristics of victims for certain types of fraud, including special fraud (i.e., crime committed through pretending to be someone special to the victim, such as a friend, a relative, or an employee; Ueno et al.) and COVID-19 scams (Nolte et al.). Lin et al. explored the neural (i.e., amygdala activation) and biological (i.e., oxytocin) mechanisms for age differences in face trustworthiness judgments (assumed to be related to fraud susceptibility).

The systematic review by Shang et al. aimed to identify common psychological characteristics of older fraud victims, in a specific context of online/Internet scams, by reviewing a selection of 21 research articles. It follows a rigorous process of literature search and article screening. The risk of bias was thoroughly analyzed. A number of general conclusions could be drawn: (1) There is no convergent evidence for a heightened prevalence of online fraud victimization among older adults than other age groups; (2) There is no consensus on the role of cognitive function, mental health, and physical health in online fraud victimization of older adults; and (3) The techniques used by fraudsters and past fraud experience might be related to older adults' fraud susceptibility. This view challenges the predominant and popular view of an aging-associated increase in fraud victimization (James et al., 2012). However, the result well aligns with some other studies (e.g., Ross et al., 2014).

The COVID-19 pandemic caused a surge in scams. Nolte et al. examined age differences across younger, middle-aged, and older adults in COVID-19 scam vulnerability, as indexed by their responses to COVID-19 solicitations (e.g., willingness/likelihood to click a link or purchase the featured product). The study also examined some psychological and sociodemographic factors related to COVID-19 scam vulnerability. No age differences were detected in the willingness to respond to scam solicitations. Nevertheless, older adults showed a differentially more cautious response tendency toward scam information. Specifically, they tended to view scams as less beneficial and more risky relative to other age groups. Furthermore, higher education, being married, past fraud experience, and higher positive urgency were identified as predictors of scam vulnerability. Finally, scam response intention was well predicted by higher perceived genuineness and benefits and lower perceived risks associated with the scam solicitations. The results suggest that scam susceptibility is more likely a result of poor impulse control. Even past scam victimization experiences would not inhibit this urge.

Using a slightly different approach, Ueno et al. compared fraud victims with non-victims of special frauds among Japanese older adults. The results identified some critical psychosocial characteristics of victimized older adults: being female, living alone, going out infrequently, being overconfident against fraud, and responding quickly to phone calls or visitors.

Taking a slightly different approach, Lin et al. examined the face trustworthiness judgments between younger and older adults and the underlying brain (i.e., amygdala activation) and biological (i.e., oxytocin) mechanisms. Specifically, younger and older adults received oxytocin or a placebo through nasal spray before the face trustworthiness rating task coupled with an fMRI scan. No overall age differences were found in the rating performance, but older adults rated ambivalent, untrustworthy-looking faces as more trustworthy relative to younger adults. The lateralized amygdala activation was differentially related to face trustworthiness ratings for younger and older adults. Importantly, the single-dose oxytocin did not modulate behavioral or brain effects involved in face trustworthiness ratings in either age group.

Taken together, this collection of articles sheds important light on our understanding of fraud susceptibility, its age differences, psychosocial and demographic predictors, and neural/biological markers. Overall, the literature on age differences in fraud victimization has not reached a clear consensus, but younger and older adults showed different neural mechanisms for fraud regulation.

## Author contributions

LY: Conceptualization, Writing – original draft, Writing – review & editing. XZ: Writing – review & editing. JY: Writing – review & editing.
